# Preventing monkeypox outbreaks: Focus on diagnosis, care, treatment, and vaccination

**DOI:** 10.1017/cts.2023.11

**Published:** 2023-02-03

**Authors:** Moslem Ghaseminia

**Affiliations:** Department of Microbiology & Virology, Faculty of Medicine, Tabriz University of Medical Sciences, Tabriz, Iran

**Keywords:** Monkeypox virus, Orthopoxvirus, antiviral drugs, vaccination, Poxviridae

## Abstract

The first human case of monkeypox virus (Mpox) was reported in 1970. In the years after 1970, human infection with Mpox and human-to-human transmission was not widely observed, and more cases were seen in endemic areas. In that year, Mpox spread was confirmed through the export of infected animals to other parts of the world. Every few years, sporadic infections were reported in different parts of the world from human contamination and human-to-human transmission. In recent years, with the slow decline of the COVID-19 pandemic, the outbreak of Mpox was observed in many countries of the world. To deal with the spread of this viral infection, we need to know the ways to diagnose the infection, treat the infection, care for the patients, and implement a wide program of vaccination. Currently, there are no specific drugs available for this virus, but according to previous studies related to smallpox, drugs such as tecovirimat, cidofovir, and brincidofovir, which were used for smallpox and other orthopoxviruses in the past, can be considered to deal with Mpox. Also, some vaccines such as JYNNEOS, IMVAMUNE, and MoVIHvax that have been used against smallpox can be useful to some extent in preventing Mpox.

## Introduction

The first human infection caused by monkeypox (Mpox) was first confirmed in 1970. Until then, outbreaks of Mpox had been observed among captive mammals. In careful laboratory investigations to find the latest cases of smallpox and people suspected of having smallpox to carry out smallpox eradication programs in West and Central Africa, several documented samples of Mpox were identified and observed [1]. With the identification of numerous new cases at that time, Mpox received more attention than before. So hundreds of other cases of Mpox were identified and confirmed by reference laboratories in Central and West African countries [2]. Although the number of monkeypox (MPX) cases was increasing, no human infection with Mpox was reported in West Africa from 1978 to 2010 [3]. However, different waves of MPX outbreaks have been recorded in human populations and different countries. After the slow decline of the coronavirus disease 2019 (COVID-19) disease in recent years, a large number of cases of MPX in humans have been identified and confirmed in different countries. The high prevalence of cases in non-endemic countries and the distance from the smallpox vaccination program have increased the concerns related to Mpox [4]. Recent reports of large outbreaks of MPX in different countries have made Mpox an emerging infectious pathogen of concern associated with significant mortality in humans [5].

In the early years of MPX outbreaks and disease in humans, epidemiological studies conducted in the Democratic Republic of the Congo (DRC) estimated an annual incidence rate of MPX of 0.63 per 10,000 people, defining MPX as a rare disease. However, recent estimates have shown that we are facing a sharp increase in the incidence of MPX, and the incidence rate has reached 5.53 per 10,000 [3,6]. The recent clinical data and the estimates made consider the mortality risk for smallpox to be small, but deaths related to MPX have been recorded in the basin and require more detailed investigations. Logically, with the increase in the number of cases of sick people, the number of deaths also increases [7]. From the time of the first reports until a few years ago, isolated and scattered cases of MPX in humans were reported. In 2022, the number of infected people suddenly increased significantly. In May 2022, many cases of MPX have been reported in Spain, Belgium, Sweden, Italy, and Portugal in Europe. Similarly, there has been a report of human infection with MPX from Canada in North America. In the same month of May 2022, two patients were identified in Australia, both of which returned to Australia from Europe (non-endemic areas of MPX). As of late May 2022, several confirmed cases of MPX have been reported from Germany, the Netherlands, and the United Kingdom (UK) [8].

Mpox, the causative agent of monkeypox disease, which is known as a zoonotic disease, is classified in the family Poxviridae and genus Orthopoxvirus. Although Mpox has been detected in different regions of the world in recent years, it is considered native to Central and West African countries. All the viruses of this family have a double-stranded DNA genome (197 KB) that encodes about 190 proteins that are responsible for the biological activities of the virus, such as escaping from the immune system, pathogenicity, and diversity in the host and infecting cells [9]. While Mpox was first identified in monkeys and given this name, it infects many species of animals. Mpox is capable of infecting a wide variety of nonhuman mammalian species, chickens, and domestic pigs, and rodents such as rats, mice, and squirrels [10]. Despite many studies on MPX and its pathogenesis in humans, many aspects related to this virus such as ways to detect the virus, precautions necessary to prevent the spread of the virus, treatment and care and reducing mortality, and immunization human population (vaccination) remain unclear. Considering the spread of this virus in non-native areas, this question is removal whether with our current experiences and knowledge about monkeypox, we can deal with the wide spread of monkeypox. The purpose of writing this study is to know the methods of diagnosis, care, and treatment of patients for current infected cases and to investigate the role of available vaccines for immunization in critical times in the future.

## Poxviruses

Viruses of the Poxviridae family are all enveloped, brick-shaped, or oval-shaped with a length of 220–450 nm and a width of 140–260 nm. The surface membrane displays surface tubules or surface filaments [11]. The combination of proteins and the coating of members of the Poxviridae family give the virion a round appearance, and it is structurally bulky. Their DNA genome is surrounded by a protein core containing a set of enzymes involved in the replication and transcription of DNA [12,13]. These enzymes include RNA polymerase, DNA polymerase, and capping enzymes for mRNA, which are unique among other viruses and enable the virus to reproduce in the cytoplasm [14]. Because of their large appearance, Poxviruses were among the first viruses to be observed under a microscope. Guarneri and Paschen intracytoplasmic inclusions can be seen in squamous and visceral cells infected with Poxviruses. These inclusions are somewhat characteristic of viruses, such as even Henderson-Peterson bodies seen in MCV [15]. Poxviruses have a complex life cycle due to their various infectious forms. Their life cycle consists of enveloped and unenveloped virions, each of which is infectious. These viruses take their lipid coating from modified Golgi membranes where specific viral polypeptides such as hemagglutinin are accumulated [16].

Orthopoxvirus, Parapoxvirus, Yatapoxvirus, and Molosipoxvirus are genera belonging to the Poxviridae family that infect humans. Smallpox virus (variola), vaccinia virus, cowpox virus, and Mpox are in the Orthopoxvirus genus, orf virus, pseudopox virus, and bovine papular stomatitis virus are in the Parapox genus, Tanapox virus and Yaba monkey tumor virus are in the Yatapox genus, and molluscum contagiosum virus (MCV) is in the Molluscipox genus, each of which is capable of infecting humans. Until the last few years, vaccinia and MCV were among the most common viruses that infect humans, but in recent years, MPX infection has been increasing in human populations [17].

Some viruses of the Poxviruses, such as variola and MCV, specifically infect humans. However, some members of the genus Orthopoxvirus are zoonotic and cause diseases in humans, such as vaccinia, cowpox, MPX, and orf [18].

For this reason, there are many studies related to the pathogenesis of smallpox and molluscum contagiosum. MCV causes common genital lesions in children and adults. Skin lesions caused by this virus in immunocompromised people become a very widespread debilitating skin disease [19].

Based on previous studies, the mortality rate of smallpox is estimated to be 10–30%, which leaves blindness and unsightly wounds in the survivors. The virus was deadly for years until the widespread use of the vaccine reduced smallpox cases and was eventually declared eradicated in 1980 by the World Health Organization [20].

Unlike smallpox and MCV, which only infect humans as their only host, Mpox as another member of this family is a zoonotic virusMpox, which received more attention after its first case was identified in humans in 1970. The increase in its spread in recent years has increased concerns after several years [8]. Many of the rodent genera suspected as possible reservoirs of the virus, including Cricetomys, Graphiurus, Funisciurus, and Heliosciurus, are found both in the forests of West Africa and the Congo Basin [21]. This diversity in the host plays a big role in the spread of the disease. In the study published by Ramnarayan et al., they reported the infection of the members of a family including a father, mother, and a 13-day-old baby. The father of this family likely contracted Mpox through a mouse killed by MPX, and he passed it on to the woman and His child [11].

## Monkeypox epidemiology

This virus was named monkeypox for the first time because the first case was observed in a macaque monkeys, and yet monkeys are not the original source of Mpox. While the original origin of Mpox is not definitively known, some small mammals and rodents have been attributed as the source of Mpox [22]. Until the spread of Mpox in recent years, it was rarely reported from human to human. However, human-to-human transmission is attributed to respiratory droplets or direct contact with mucous skin lesions of infected individuals [23].

Since May 7, 2022, more than 100 human cases of MPX have been reported in more than 100 countries worldwide, making it the largest outbreak of MPX recorded to date. Manifestations of MPX disease are similar to smallpox, but less severe [24].

Despite the 2017 outbreak of MPX in Nigeria including many cases in savannah and urban areas, epidemiologic studies have shown that African rainforests are among the main reservoirs of the Mpox [25].

According to studies published in 2003, the first outbreak of MPX in the USA was reported after rodents were imported as pets from Ghana, and eventually, 47 confirmed cases were identified and reported that year [26].

According to this case and other reported cases of the export of animals such as monkeys to non-endemic countries of MPX, most attention was paid to zoonotic transmission of this virus and human-to-human transmission was very limited [27]. However, in September 2018–2019 in the UK, after the identification of three people infected with MPX who had a history of travel to Nigeria, it was observed that a human-to-human hospital transmission and a family contagion occurred after hospitalization [28].

These observations and the widespread of MPX in other parts of the world largely justify human-to-human transmission in MPX patients and will increase concerns. In most cases, there is no epidemiological link between patients infected with MPX and Africa, and it can be inferred that human-to-human transmission has caused the spread of the virus in non-endemic areas [29].

Mutations and differences in strains cause epidemiological changes and changes in the spread of the virus. The pathogenic behavior of both types of Mpox identified in Central and West Africa has been investigated in different species from rodents to primates. It has been reported that there are tangible differences between acuteness (LD50), the mortality ratio, and the rate of systemic release [3,30]. To date, two main strains of the Mpox have been identified in Central and West Africa, with the strain identified in Central Africa being associated with a more severe disease [31]. Unlike the Central African strains, the strains observed in West Africa had a lower tendency to spread from person to person. In general, genetic changes and the emergence of diverse species lead to obvious geographical differences in the prevalence of MPX [32].

## Diagnosis, care, and treatment

### Diagnosis

Early detection of infectious agents such as viruses is one of the priorities of health and care organizations. Regarding virus infections, it is very important to find virus detection methods in controlling the epidemic and helping to treat patients [33]. Today, many diagnostic methods are used to identify the viral agents that cause the disease. Enzyme-linked immunosorbent assay (ELISA), in situ hybridization, polymerase chain reaction (real-time PCR), reverse transcription polymerase chain reaction (RT-PCR), electron microscope, investigation of cytopathic effects (CPE), and biosensors are routinely used for detection worldwide [34,35]. During the COVID-19 pandemic, the need for fast and accurate virus detection methods was felt more than before. For this reason, many studies were conducted in connection with the identification of various markers for early and accurate identification of COVID-19 [36,37]. However, methods such as cell culture and electron microscopy are still used and important in the preparation of vaccines. Today, viral culture has largely been replaced by nucleic acid amplification tests and antigen detection techniques.

In viral infections, whose clinical symptoms are close to other diseases, especially infectious and viral diseases, the role of accurate diagnosis methods is more apparent. The clinical manifestations of MPX in humans are close to chickenpox (Varicella-Zoster Virus (VZV)), and at least in children, it should be differentiated from chickenpox. After being exposed to the sick person, symptoms appear on average 8–12 days later. The first clinical signs of MPX are fever, headache, lymphadenopathy, and flu-like symptoms. In MPX disease, like chickenpox, there are also reports showing that fever and skin rashes are visible and affect most areas of the body, including the palms and feet [38]. Monkey pox disease is considered as a self-limiting disease, but in pregnant women, children and immunocompromised people, it may occur as a more severe disease [39]. In some studies, it has been stated that MPX can be divided into two stages. The incubation period of the disease is approximately 7–13 days, and in the first stage, a multi-day prodromal period begins with fever, lymphadenopathy, similar to encephalitis, and the second stage is associated with skin lesions. Skin lesions usually start as macular and go through papular, vesicular, pustular, and umbilical stages. The rash usually starts on the face and changes to the palms and soles of the feet. In some cases, the oropharyngeal mucosa is involved and painful [40,41]. Most skin lesions in the current outbreak of 2022 have been observed in the genital area or perineum [42].

As with electron microscopy, culturing viruses for detection is associated with limitations and difficulties. Finding suitable cells for virus replication, virus isolation, and the need for a laboratory with appropriate equipment are among these. However, CPE screening methods are used for some viruses. Some studies have reported that Mpox grows rapidly in cell culture and produces detectable CPE in RMK, BGM, A549, and MRC-5 cell lines in approximately 2 days [43]. However, methods such as cell culture and electron microscopy are still used and important in the preparation of vaccines. Today, viral culture has largely been replaced by nucleic acid amplification tests and antigen detection techniques.

The clinical manifestations of MPX in humans are close to chickenpox, and at least in children, it should be differentiated from chickenpox. The first clinical signs of MPX are smallpox, fever, headache, and prostration, and after being exposed to the sick person, symptoms appear on average 8 to 12 days later. In smallpox, there are also reports showing that fever and a skin rash are visible and affect most areas of the body, including the palms and soles [44].

Clinical manifestations in MPX are not specific, and in many viral diseases and even other nonviral infections, these clinical signs and symptoms can be observed. In some cases, especially early in the onset of clinical symptoms, this issue can make it difficult for doctors to distinguish MPX from other viral, bacterial, or other diseases with similar symptoms. Therefore, accurate and fast laboratory diagnosis methods are of particular importance in such conditions [45]. Peiró-Mestres et al have traced MPX DNA in different samples from 12 patients. In total, they observed MPX DNA in saliva, rectal swab, nasopharyngeal swab, semen, urine, and feces samples. In all 12 patients, the saliva sample contained MPX DNA. This shows the importance of saliva sampling for laboratory diagnosis of the virus. They have also reported that in rectal swab samples (11/12 cases) and nasopharyngeal swab samples (12/10 cases), the most identified cases were after saliva [46]. However, many other studies emphasize the detection of Mpox DNA in skin rashes. In any case, this study confirms the presence of the virus in different samples, and in this way, the virus can be spread to others [46].

The samples obtained from skin lesions, throat, blood, and urine of a patient suspected of having Mpox can be performed with real-time PCR technique after DNA extraction. Real-time PCR has high sensitivity and specificity for detecting Mpox and other viruses. It is difficult and expensive to equip all laboratories, especially in developing countries. However, the detection of viral DNA in swabs taken from wounds and skin lesions of patients is the main strategy for diagnosing suspected cases of MPX [31]. In the diagnosis of MPX, clinical and laboratory tests should be used, and MPX should be differentiated from other skin infections that cause rashes, such as chickenpox, measles, bacterial skin infections, scabies, syphilis, and even sensitivity to foreign substances. Lymphadenopathy is one of the clinical manifestations of MPX that helps distinguish MPX from chickenpox. However, the infection can be confirmed by detecting the DNA of the virus. PCR testing of skin lesion samples is preferable to PCR of blood samples in diagnosing Mpox. Because the virus only stays in the blood for a short time, detecting Mpox DNA in the blood is not definitive. Also, information such as the time of onset of fever, the onset of skin lesions, the appearance of skin rashes, and the history of travel or contact with a dead animal can help interpret the test results [45,47]. Despite the recent increase in cases and with the increase in the number of people who have not traveled to endemic areas, it is useful to consider this issue and check the international travel status of patients or people who have been in contact with them in the 21 days before the onset of symptoms[48].

### Treatment

The importance of antibody responses in protecting patients from Poxvirus infections has been demonstrated in various studies [49,50]. To date, the Centers for Disease Control and Prevention (CDC) has emphasized only supportive care and has not announced a specific treatment for MPX, and antiviral drugs for MPX have not received final approval [51]. Supportive treatments such as maintenance of fluid and electrolyte balance, nutrition, symptomatic treatment with antipyretic/analgesic drugs, early identification of secondary infections, and prompt treatment with appropriate antimicrobial agents are recommended for the treatment of MPX disease [52]. Some treatments that were previously used to control and treat smallpox patients are also being investigated in some studies for their effectiveness in the treatment and control of MPX disease.

Tecovirimat, available as an oral capsule or an intravenous vial, is considered the first antiviral drug to treat smallpox. The new protocol maintained by the CDC allows emergency use of tecovirimat for non-variola infections such as patients with MPX [53]. Tecovirimate has an inhibitory effect on p37, which is a highly conserved protein among orthopoxviruses, preventing the formation of virions and their exit from the cell. Finally, tecovirimate prevents the virus from spreading in the body by preventing the virus from leaving the infected cell [54].

So far, there is no approved antiviral treatment for MPX. Nevertheless, due to the small number of deaths reported due to this disease and the severity of the disease in some people, immunocompromised people, and pregnant women, antiviral treatment should still be one of the research options [55]. Currently, clinical studies are being conducted to investigate the effectiveness of this drug on MPX patients (Table 1).

**Table 1.** Some ongoing studies to investigate the effectiveness of tecovirimat in the treatment of monkeypox

NCT number	NCT05559099	NCT05534984	NCT05534165
Phase	2	3	3
Dose	3 to <6 kg: 100 mg; 6 to <13 kg: 200 mg; 13 to <25 kg: 400 mg; 25 to <40 kg: 800 mg; 40 to <120 kg:1200 mg; ≥120 kg: 1800 mg	25–40 kg: 400 mg every 12 h – 14 D; 40–120 kg: 600 mg every 12 h – 14 D; 120 kg and over: 600 mg every 8 h – 14 D	600 mg – two times a day
Participants	450	530	120
Ages/sex	Child, adult/all	Child, adult/all	Adult/all
Inclusion criteria	Confirmed by PCR – patient has at least one active – nonpregnant – weight ≥ 3 kg	Confirmed or presumptive HMpox infection; HMpox illness of <14 days; at least one active (not yet scabbed); nonpregnant people	Weight ≥ 40 kg, confirmed by PCR, culture, or antigen test, skin lesion(s), mucosal lesion(s)
Recruitment status	Recruiting	Recruiting	Not yet recruiting
Responsible party	NIAID/USA	NIAID/USA	McGill University/CANADA

NTC number, National Clinical Trial; kg, kilogram; mg, milligram; D, day; h, hour; PCR, polymerase chain reaction; HMpox, human monkeypox virus; NIAID, National Institute of Allergy and Infectious Diseases.

Cidofovir was used in medicine in 1996 as an injectable and topical antiviral drug mainly for the treatment of retinal infection caused by cytomegalovirus (CMV) in HIV-infected patients [56]. It has been shown in laboratory conditions that cidofovir may have an anti-smallpox effect and can be used in emergencies such as bioterror with smallpox. However, due to its abundant nephrotoxicity, it has been used less. Brincidofovir is a modified form of cidofovir that has greatly reduced renal toxicity. Brincidofovir is taken orally with higher inhibitory activity than cidofor against smallpox [57,58]. Brincidofovir is derived by conjugation with a lipid molecule to cidofovir. This binding and finding the lipid side of the drug facilitates binding to the cell membrane, and then entering the cells. When the drug enters the infected body, cidofovir is separated from the drug, phosphorylated, and converted into its active form. This drug inhibits the synthesis of viral DNA that is carried out by the intermediary of viral DNA polymerase. It can also act as a noncyclic nucleotide and attach itself to viral DNA and in this way prevent viral DNA synthesis and virus multiplication [59].

As of June 2021, brincidofovir has been approved for the treatment of smallpox in the USA [60]. Many studies related to the effectiveness of brincidofovir and cidofovir in the treatment of MPX are not available; however, some studies have shown that brincidofovir can be effective against orthopoxvirus infections [60,61]. It should also be noted that improper use of these drugs may cause drug-resistant strains. Smee and his colleagues have stated in their study that there is a possibility of the emergence of cidofovir-resistant human orthopoxvirus infections that cannot be treated with cidofovir. More studies on the cidofovir-resistant Mpox are needed to confirm this issue [62]. To date, little evidence supports antiviral therapy with cidofovir and even tecavirimate for the treatment of severe cases of MPX [63]. However, some studies have used cidofovir to treat severe cases of monkeypox, despite the small number of patients. In Raccagni et al.’s study, no side effects and no significant changes in creatinine levels were reported, and they stated that no new symptoms were observed in patients after cidofovir administration [64].

Many studies have been conducted to investigate the effectiveness of immunoglobulin therapy on viral infections. Until today, many cases of success of treatment with immunoglobulins in the control and treatment of viral diseases have been recorded and reported. Nowadays, antibodies are prescribed routinely for some viral diseases. For example, when people who have been bitten by a dog go to the hospital, according to approved protocols, anti-rabies immunoglobulins are prescribed and injected after primary care [65,66]. The FDA has approved the use of vaccinia immune globulin intravenous (VIGIV) for the treatment of complications from vaccinia vaccination. CDC has recommended a protocol that can be used to treat orthopoxviruses such as MPX in an outbreak of VIGIV. However, there is still no information available about the effectiveness of VIGIV for the treatment of MPX. Therefore, no proven benefit of using VIGIV in MPX patients in preventing severe infection has been documented. It is recommended that VIGIV be used in persons with severe immunodeficiency in T-cell function (individuals with contraindications to smallpox-attenuated virus vaccine) who have been exposed to a sick person [52]. In some studies, it has been shown that in the animal model of rhesus macaques, anti-vaccinia antibodies alone cannot prevent death caused by Mpox infection [67]. In other viral infections such as influenza and poliovirus, adequate amounts of antibodies induced by vaccination with killed virus vaccines have been shown to prevent severe diseases caused by influenza and polioviruses [68]. The prerequisite for using antibodies is knowing the immunogenic identity of viral antigens. The identity of vaccinia immunogens is still not fully known to extract effective antibodies against Poxviruses, especially MPX. However, some studies have reported some degree of protection against the lethal Mpox challenge by antibodies raised against a single IMV immunogen (L1R). The combination of EEV immunogens (L1R, A27L, A33R, and B5R) with IMV immunogens (A27L, L1R) has also been reported to provide acceptable protection against severe MPX disease. These antigens can be the target of various studies to produce vaccines and antibodies to protect against Mpox and other members of the orthopoxvirus genus [69].

### Care

Due to their unique capabilities, viruses can create widespread epidemics. However, there are useful and reliable methods that, if used and followed properly, can prevent the occurrence of an epidemic or the further spread of the virus in society [70]. Applying methods to control and prevent the transmission of viruses in diseases that are capable of human-to-human transmission is more tangible than other viruses such as zoonotic viruses. Many studies have reported that patients with MPX in their study did not have a history of traveling to endemic areas or being exposed to an infected animal. This emphasizes human-to-human transmission [71]. Our experience with the respiratory pandemic of COVID-19 emphasizes the use of precautions and the application of appropriate methods to prevent the further spread of viral infections. In the face of COVID-19, many countries used non-pharmacological interventions (NPIs) to reduce the spread of the disease, such as social distancing, school closures, “testing and isolating” symptomatic individuals, hand hygiene, respiratory etiquette, and environmental cleaning. The use of these methods not only played a role in the spread of COVID-19 but also affected several infectious diseases based on studies [72].

To overcome the spread of MPX, it is necessary to know the methods of transmission and pathogenesis of this virus. Our understanding of the tropism and pathogenicity of Mpox in humans is not complete, and most of what we know has been obtained from studies on animal models and studies on other orthopoxviruses. For example, in an animal model by studying macaques, it has been shown that these animal models are easily infected by the mucous membranes of the mouth and respiratory tract when exposed to aerosols, and the primary target of infection is the epithelial cells of the airways [73].

It has been observed that in addition to skin lesions, secondary complications of infection such as gastroenteritis, pneumonia, encephalitis, and secondary bacterial skin infections have also occurred in people, which in addition to the severity of the disease, and increases the chance of transmission [74]. Although smallpox infection during the outbreak in the USA was assessed as a mild infection, reports of more severe manifestations involving the central nervous system, gastrointestinal tract, and respiratory tract are available from patients in the Congo region, indicating the diverse behavior of the virus. For example, in a study that evaluated 34 MPX patients, it was reported that nearly a third of the patients were hospitalized, and half of the children in this study were hospitalized in the intensive care unit. It is also reported in this study that patients with hypoalbuminemia followed by loss of digestive fluids had more severe disease manifestations [75]. Various studies have been conducted to identify the ways of Mpox transmission. In addition to respiratory transmission and direct transmission through skin lesions, some studies have reported the detection of the virus in the semen of patients. This issue and the creation of multiple skin lesions in the genital area can strengthen transmission through sexual intercourse among people [76]. In addition to the transmission through direct contact with lesions, it is transmitted through fomites or indirect contact with lesion material, such as virus-contaminated substrates. For this reason, in cases of hospitalization of patients, risk factors of transmission such as using the same equipment as an infected person and using a shared room and shared bed should be considered [77].

Due to the zoonosis of Mpox and people’s acceptance of pets, the possibility of disease transmission through pets should also be considered. The possibility of transmission of Mpox from humans to pets and vice versa is still not well understood. However, some studies have confirmed and reported evidence of transmission of Mpox from humans to dogs [78]. In studies published in the past years, it has been reported that wild animals such as rodents and primates are carriers of Mpox in endemic areas [79]. However, there are some reports of prairie dogs being infected in the USA and of caged mammals that have been in contact with imported infected animals infected with Mpox in Europe [80,81]. In some studies, it has been reported that in some animals with monkey pox, the infection is asymptomatic. The wide range of species infected with Mpox and its wide host range show that most animal species can be infected with Mpox and should be considered important in the study of the epidemiological cycle of Mpox [82].

In the current outbreak of MPX in 2022, it has been observed that many infected individuals who are infected with the Mpox are homosexual and bisexual men [51]. Despite all the mentioned cases, the transmission of Mpox to a person requires long-term contact with the source of infection or the sick person. In cases where a person has to be exposed for a long time (healthcare workers), the need for more monitoring and getting a vaccine as post-exposure prophylaxis (PEP) has been recommended. Among the cases of contact with a high chance of transmission, the following can be mentioned: 1 – Traveling and working in the patient’s room without using an N95 mask and eye protection. 2 – Repeated unprotected contact (without gloves) with the patient’s skin lesions. 3 – Sex and contact with body fluids such as the patient’s saliva. 4 – Contact with contaminated items and materials such as the clothes of an infected person [60]. Orthopoxviruses maintain their infectivity on inanimate surfaces for up to 42 days. As a result, observing and applying disinfection methods plays an important role in controlling the transmission of these viruses. Seventy percent ethanol (≤1 min), 0.2% peracetic acid (≤10 min) and 1–10% a probiotic cleaner (1 h), sodium hypochlorite (0.25–2.5%; 1 min), 2% glutaraldehyde (10 min ), and 0.55% ortho-phthalaldehyde (5 min) has the ability to inactivate orthopoxviruses [83].

## Vaccination

The first attempts at immunization are documented in the 4th century in China and India, which attempted to immunize healthy people against smallpox by inoculating a milder form. Also, in 1786, Edward Jenner, after observing a mild disease of smallpox in dairy cows previously infected with cowpox, concluded that the use of cowpox can provide immunity against smallpox [84]. Several studies have been conducted in the past that have shown that people vaccinated with the smallpox vaccine are somewhat safe from MPX, and the disease caused in these people is much milder and the manifestations of the disease are very insignificant [85]. These data show that finding an effective vaccine against smallpox, in addition to being effective in the MPX epidemic, will also help public health in the event of bioterrorism and intentional smallpox outbreaks [67,86]. Based on these data and documents, people vaccinated against smallpox at the time of MPX infection have less severe disease and have shown better protection against MPX [87]. This issue and other documents since the time of Edward Jenner show that antibodies produced against viruses of this family can have a cross-effect. The same principles that Edward Jenner used from cowpox to vaccinate against smallpox [88]. If this hypothesis is confirmed, it will be possible to protect communities against MPX and the dangers of smallpox bioterrorism by designing a vaccine at the same time. To confirm this issue, extensive studies are needed to confirm the effectiveness of available vaccines against different viruses of the pox family. With the outbreak of MPX, studies can be designed and helpful results can be obtained and hit two targets with one arrow.

The majority of representatives of the World Health Assembly at the 64th meeting of this organization in May 2011 emphasized the elimination of the remaining stocks of variola virus. But it should be noted that by removing the storage source, there will be problems for conducting studies [3]. Due to the risks and possibilities of intentional use of smallpox, it has been reported that vaccination of a part of the population in the USA has been done, observing few cases of side effects in some people, such as people with immunodeficiency, who have not been vaccinated. Also, the USA stopped mass vaccination in the early 1970s due to the observation of some complications and that the side effects outweighed the benefits in the absence of the smallpox threat [67].

After smallpox was eradicated, smallpox vaccines were still being researched. Even the development of The Dryvax smallpox vaccine, which contains a live attenuated virus, was not well received by the public or the scientific community [5]. Considering the eradication of smallpox and the lack of widespread use of smallpox vaccines, it seems that the best option, considering the current conditions and the spread of MPX in many parts of the world, as well as the risk of the reappearance of smallpox caused by the vaccine, is to focus on production. And research in the field of MPX vaccines should be focused. Currently, several types of researches are being conducted in the field of making and evaluating the efficacy of anti-monkeypox vaccines (Table 2).

**Table 2.** Clinical studies related to different vaccines and their effectiveness in preventing monkeypox

NCT number	NCT02977715	NCT05512949	NCT05562323
Vaccine name	IMVAMUNE	JYNNEOS (MVA-BN)	MoVIHvax
Type of vaccine	Attenuated live virus smallpox vaccine	Highly attenuated strain of the poxvirus Chorioallantois Vaccinia virus Ankara	Live Modified Vaccinia Virus Ankara
Phase	3	2	Cohort/prospective
Participants	1600	210	100
Ages/sex	18 years and older/all	18–50 year/all	18 years and older/all
Inclusion criteria	Males and nonpregnant females; healthcare personnel at risk of monkeypox infection	Nonpregnant; stable state of health	HIV-negative and HIV-positive individuals
Recruitment status	Active, not recruiting	Recruiting	Recruiting
Responsible party	CDC	NIAID	The Fight AIDS Foundation

NTC, National Clinical Trial; MVA-BN, Modified Vaccinia Ankara-Bavarian Nordic; HIV, human immunodeficiency virus; CDC, Centers for Disease Control and Prevention; NIAID, National Institute of Allergy and Infectious Diseases.

Because smallpox vaccines are live attenuated vaccines, the use of these vaccines in immunocompromised individuals such as acquired cellular immunodeficiency caused by HIV-1, aggressive chemotherapy for cancer, and organ transplants may be associated with severe and sometimes fatal complications. However, based on what we said earlier, HIV-infected people, such as gay men, are more at risk of MPX infection. However, some vaccines are also being investigated to determine their effectiveness and efficiency in these people (Table 2, NCT05562323). However, Dryvax as an existing approved vaccine can cause problems in immunocompromised people [89]. Despite the stated risks of the Dryvax vaccine for immunocompromised individuals, this vaccine has been shown to be protective against MPX disease and infection after an intravenous challenge [90].

One of the obstacles facing the development of vaccines is the lack of animal models. This seems to have been less challenging with MPX. In a study, Hooper et al tried to investigate the effectiveness of a smallpox DNA vaccine against MPX. They have reported that infection with the Mpox in a rhesus macaque model caused a disease similar to human pox, and this animal model can be well used in vaccine studies for the Poxviruses [91]. Also, this animal model is a suitable model for studies in the field of vaccination and immune response to the vaccine because it has the ability to quantitatively measure Mpox genomes in blood, tissues, and skin in artificial MPX infection [67]. Vaccines developed for protection against MPX are capable of providing lasting immunity with a single dose and are prescribed to high-risk individuals exposed to sources of contamination [3]. This seems to indicate the greater importance of humoral immunity in vaccinated individuals. However, to defend against natural infections with viruses, cellular immunity plays a prominent role. Also, monocytes from Mpox-infected individuals have been shown to have a higher ability to stimulate T cells to present Mpox antigens than from vaccinated individuals [92]. Decreased immunity is known as one of the reasons for the emergence and increase of viral infections.

Nowadays, researchers consider one of the important factors in the emergence of MPX to be the reduction of the immunity of society, especially in young people. Despite the effects of smallpox vaccination on immunization against MPX, previous outbreaks of MPX in the USA found that smallpox vaccination did not provide complete immunity against MPX. It is said that one out of five confirmed patients has already received this vaccine [75]. Modified Vaccinia Ankara (MVA) is an attenuated strain of vaccinia that does not undergo a complete replication cycle in mammalian cells. However, after intramuscular (IM) injection in two doses, it has been observed that MVA is immunogenic and protects nonhuman primates against this virus. It is also reported that the immunity created against MVA lasts for more than 2.5 years [91]. Some viruses become weakened strains for vaccination by exposure to certain chemicals or physical methods. These vaccines lose part of their virion structure or functional enzymes; hence, they appear weakened compared to the wild strain. There is another vaccine called LC16m8 which is not able to express the functional B5R gene. This product of this gene is related to actin mediators and plays a role in cell-to-cell virus transmission. LC16m8 strain from nonhuman primates has been observed to perform well against reducing the clinical manifestations of MPX [93].

As we mentioned earlier, the use of live vaccines is associated with challenges such as not using these vaccines in immunocompromised people. Another challenge for live vaccines such as MVA and LC16m8, which are both components of live vaccines, is their preparation and storage conditions. To solve this problem, LC16m8 is prepared and distributed as freeze-dried, and unlike MVA, it does not need cold storage. The need for a cold transfer chain for MVA, especially in African countries, is one of the challenges of this vaccine [94]. Today, next-generation vaccines based on DNA or purified immunogenic proteins can help to overcome the need for the cold chain of vaccines. Some studies to obtain vaccines with subunits of outer membrane proteins of vaccinia are in preliminary investigations. These vaccines have sometimes been proposed in combination with DNA-based vaccines to prevent fatal MPX infection in nonhuman primates [95,96]. By inferring the mentioned materials, it is easy to understand the immunity created against other genera of orthopoxviruses by the vaccine made for another genus. For example, it has been reported that in the few years after the end of routine vaccination for smallpox, it prevented up to 85% of severe diseases among family members exposed to MPX [97]. Perhaps due to this close relationship and immunity created for different viruses in the same genus, it is possible to use other viruses of this genus in the line of research related to dangerous and deadly viruses of this family, such as smallpox. Although smallpox is an exclusively human disease and MPX is a shared disease between humans and animals, studies have shown that the use of different harvests in the treatment of smallpox can be beneficial for the treatment of MPX [98]. Today, at least until the availability of a vaccine and a specific drug for MPX, the treatments and vaccines used for smallpox are suggested to prevent or treat MPX [3].

The importance of neutralizing antibodies has been confirmed in many studies. However, the immune system uses both humoral and cellular immunity to fight viruses. According to the report of previous studies, antibody responses decrease after a few years, so it is necessary to carry out studies on the effectiveness and stimulation of cellular immunity and the creation of memory cells through the vaccine. Some animal studies have shown that DNA-based vaccines can induce cellular immune responses to some extent [96,99,100]. Some studies favor an antibody response and antibody-mediated protection over class I or class II histocompatibility-limited immune responses and have reported that CD4+ and CD8+ T cells are not as essential as antibodies to protect against disease [67]. The development of a suitable DNA vaccine for MPX, like other vaccine development platforms, has its hurdles. However, in some past studies in nonhuman primates, it has been reported that the DNA-based vaccine had less humoral immunity compared to the live virus [101].

One of the challenges facing DNA vaccines is the delivery of antigens (vaccine injection). In the study of Hirao et al., the delivery of viral antigens through the intradermal (ID) route, which induces cell responses mainly of the TH2 type, has been compared with the traditional IM route. They have reported that injection and delivery of antigen from the ID pathway confer protection against lethal MPV challenges in nonhuman primates [96]. In this study, they reported that the response to IFN-γ and CD4 + T cells was greater in IM injection than in ID injection. Nonetheless, they did not report a difference in CD8+ responses between ID- and IM-vaccinated groups [96]. Due to differences in vaccine administration, these responses cannot be related, as other studies have reported that injection of ID-designed DNA vaccine induced a quadrivalent HIV response [96,102]. Furthermore, studies have shown that the use of antigens in combination with adjuvants gives better results in achieving strong antibody responses [103]. Also, new technologies to obtain new vaccines can be very helpful in this matter. In Shinoda et al.’s study, it has been reported that a slight modification in the structure of the L1 antigen in the DNA vaccine used against smallpox can achieve a stronger neutralizing antibody response [104]. Among the viruses of the smallpox virus family, several immunogens have been identified. The smallpox virus L1 protein is one of the best-known smallpox immunogens, which has been the subject of numerous studies. This protein is encoded by L1R. To date, several DNA vaccines for the L1 immunogen have been designed and tested in mice and nonhuman primates. These vaccines have been able to produce neutralizing antibodies in animal models and play a role in protecting them against fatal diseases [105].

In order to achieve an effective vaccine against Mpox, many studies need to be done to develop new vaccines and different methods of antigen delivery. In order to be successful in creating long-term immunity, it is necessary to strengthen the memory stimulation responses caused by the vaccine. This phenomenon was identified and confirmed in the study of several people who were exposed to the Mpox but did not get sick [106].

In addition to the mentioned cases, another challenge facing vaccine preparation is the strategies of escaping the immune system by viruses. Orthopoxiruses use several different methods to hide from the immune system’s detection structures or inhibit the functional structures of the immune system. It has been shown that in lymphocytes infected with Orthopoxiruses, some viral proteins suppress apoptosis in these cells by targeting innate immunity [107,108]. It has also been reported in some studies that Orthopoxiruses inhibit type 1 interferon, some pathways of the complement system, and the production of host chemokines through changes in nuclear factor-kappa B (NF-κB) [109,110].

## Discussion

More than 200 years ago, Edward Jenner observed the protection of some milkmaids against smallpox, after being infected with cowpox, and the first concepts for immunization against smallpox using cowpox were created [20]. Since the eradication of smallpox, there have been concerns about the re-emergence of this devastating disease, which has increased after September 11, 2001 [88,111]. Although MPX is a milder disease than smallpox and has a low mortality rate, it should be considered a health threat. In the past, MPX was common in endemic countries for many years, with few outbreaks reported in non-endemic countries each year. In 2022, MPX has spread rapidly in many countries. Actions such as awareness of care, treatment, diagnosis, and vaccination for Mpox are needed to overcome the further spread of Mpox worldwide.

Laboratory diagnosis methods such as ELISA and real-time PCR are used to speed up the diagnosis of patients to prevent the spread of Mpox. Using laboratory techniques to differentiate MPX from other patients such as chickenpox helps specialists. In the past, drugs such as tecovirimat, cidofovir, and brincidofovir have been used to treat smallpox, which can also be effective in the treatment of MPX. After identifying and during the treatment of patients with MPX, it is necessary to take care to prevent others from getting infected. Finally, vaccination against MPX, like other viral infections, is the best option to prevent the spread of infection in large communities. Various vaccines to prevent MPX such as IMVAMUNE, JYNNEOS, and MoVIHvax are being investigated. However, Mpox is circulating rapidly in different countries and needs more attention and more studies to understand the characteristics of the disease in order to control the disease.
